# Expression of *cagA*, *virB/D* Complex and/or *vacA* Genes in *Helicobacter pylori* Strains Originating from Patients with Gastric Diseases

**DOI:** 10.1371/journal.pone.0148936

**Published:** 2016-02-11

**Authors:** Andrzej Szkaradkiewicz, Tomasz M. Karpiński, Krzysztof Linke, Przemysław Majewski, Dorota Rożkiewicz, Olga Goślińska-Kuźniarek

**Affiliations:** 1 Department of Medical Microbiology, University of Medical Sciences in Poznań, Wieniawskiego 3, Str., 61–712, Poznań, Poland; 2 Department of Gastroenterology, Human Nutrition and Internal Diseases, University of Medical Sciences in Poznań, Przybyszewskiego 49, Str., 60–355, Poznań, Poland; 3 Department of Clinical Pathomorphology, University of Medical Sciences in Poznań, Przybyszewskiego 49, Str., 60–355, Poznań, Poland; 4 Department of Paediatric Infectious Diseases, Medical University of Białystok, University Children’s Hospital, Waszyngtona 17, Str., 15–274, Białystok, Poland; Monash University, AUSTRALIA

## Abstract

In order to better understand pathogenicity of *Helicobacter pylori*, particularly in the context of its carcinogenic activity, we analysed expression of virulence genes: *cagA*, *virB/D* complex (*virB4*, *virB7*, *virB8*, *virB9*, *virB10*, *virB11*, *virD4*) and *vacA* in strains of the pathogen originating from persons with gastric diseases. The studies were conducted on 42 strains of *H*. *pylori* isolated from patients with histological diagnosis of non-atrophic gastritis—NAG (group 1, including subgroup 1 containing *cagA*^*+*^ isolates and subgroup 2 containing *cagA*^-^ strains), multifocal atrophic gastritis—MAG (group 2) and gastric adenocarcinoma—GC (group 3). Expression of *H*. *pylori* genes was studied using microarray technology. In group 1, in all strains of *H*. *pylori cagA*^+^ (subgroup 1) high expression of the gene as well as of *virB/D* was disclosed, accompanied by moderate expression of *vacA*. In strains of subgroup 2 a moderate expression of *vacA* was detected. All strains in groups 2 and 3 carried *cagA* gene but they differed in its expression: a high expression was detected in isolates of group 2 and its hyperexpression in strains of group 3 (hypervirulent strains). In both groups high expression of *virB/D* and *vacA* was disclosed. Our results indicate that chronic active gastritis may be induced by both *cagA*^+^ strains of *H*. *pylori*, manifesting high expression of *virB/D* complex but moderate activity of *vacA*, and *cagA*^-^ strains with moderate expression of *vacA* gene. On the other hand, in progression of gastric pathology and carcinogenesis linked to *H*. *pylori* a significant role was played by hypervirulent strains, manifesting a very high expression of *cagA* and high activity of *virB/D* and *vacA* genes.

## Introduction

The causal relationship between bacterial pathogen of *H*. *pylori* and various forms of gastric disease, including gastric cancer (GC) has been well known. Pathogenicity of the bacteria is significantly related mainly to cytotoxin-associated protein A (CagA), encoded by the frequently regarded to represent virulence marker *cagA* gene, located within the pathogenicity island, *cag* (*cag*PAI) and vacuolating cytotoxin (VacA), encoded outside the *cag* PAI [1–4]. The two genes are present in genomes of around 60% strains [5]. Functional role of CagA and VacA was subjected to numerous studies [5–10]. CagA, peptidoglycan and possible other bacterial factors become translocated to epithelial cells with mediation of type IV secretion system–T4SS, in contrast to cytotoxin VacA, belonging to the group of autotransporter proteins [4, 5]. The typical secretory apparatus type IV involves a syringe-like structure, coded by *cagPAI* virulence (vir) genes *virB* and *virD4* [11, 12]. Products of the seven genes (*virB4*, *virB7*, *virB8*, *virB9*, *virB10*, *virB11* and *virD4*) compose the so called VirB/D complex, forming secretory apparatus type IV (T4SS), recognised in *Agrobacterium tumefaciens* and *Bordetella pertussis* [13]. The VirB/D complex is indispensable to translocate bacterial toxin to eukaryotic cells [14].

Therefore, it is possible that activity of genes coding for cytotoxins *CagA* and *VacA* as well as components of virB/D secretory apparatus determine a specific gastric pathology. Expression of *H*. *pylori* genes in gastric mucosal biopsies was assessed earlier [15, 16]. However, until now no efforts were made to characterize activity of specific *H*. *pylori* virulence genes, related to the progressive damage in gastric mucosa, linked to the pathogen, and to development of cancer.

Considering the above, this study aimed at analysis of gene expression manifested by *cag*PAI (including *cagA* and *virB/D* complex: *virB4*, *virB7*, *virB8*, *virB9*, *virB10*, *virB11*, *virD4*) and by *vacA* in *Helicobacter pylori* strains isolated from patients with non-atrophic gastritis, atrophic gastritis or gastric cancer.

## Materials and Methods

### Patients and *H*. *pylori* strains

The studies included 42 *Helicobacter pylori* strains, isolated from patients in the period of 2013–2014 and stored frozen at -80°C in the tryptone soya broth medium, containing 15% (vol/vol) glycerol. The excluding criterion involved use of anti-inflammatory drugs or antibiotics in the preceding 2 weeks. The patients underwent diagnostic procedures in the Department of Gastroenterology, Human Nutrition and Internal Diseases, Poznań University of Medical Sciences, Poland. All the research protocols were reviewed and approved by the Ethics Committee by the University of Medical Sciences in Poznań, Poland (No 499/15). Patient’s consent to participate in this study was obtained prior to enrolment. All participants in this study gave their written informed consent.

From patients who underwent routine gastroscopy at least four specimens (two antral and two from the corpus) were taken. Gastric biopsies were histopathologically diagnosed by analysis of hematoxylin- and eosin-stained preparations. Classification of gastritis followed the Sydney System [17]. Diagnosis of gastric cancer was confirmed histologically, evaluating advancement of the neoplastic process (staging) and grade of differentiation (grading), according to WHO classification [18].

**Fig 1:**
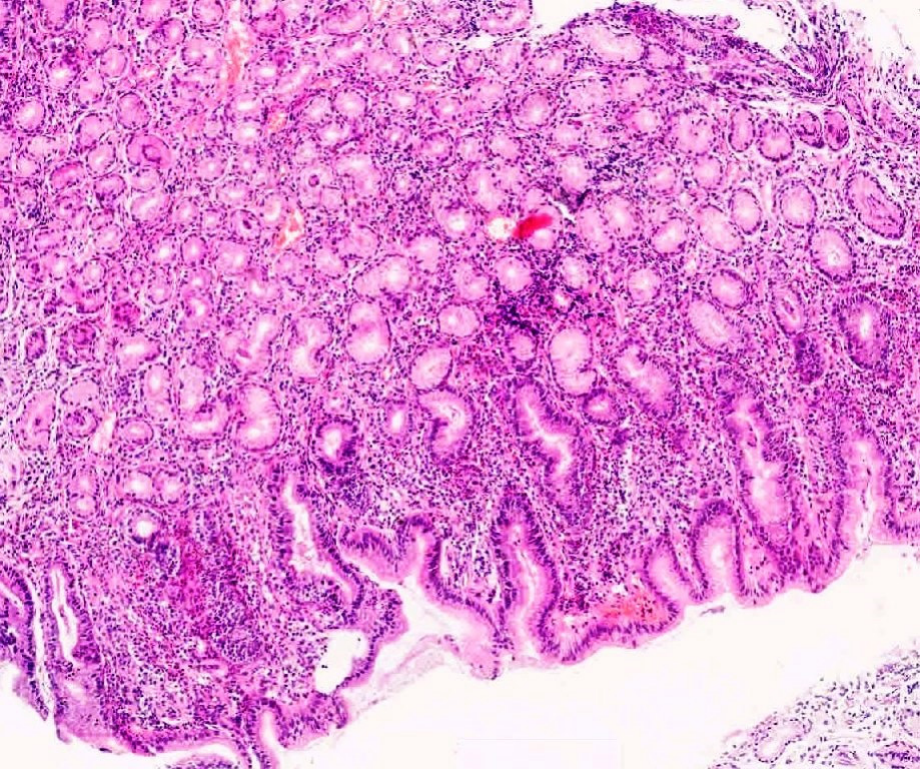
Nonatrophic superficial chronic gastritis–NAG (H&E—original magnification 40x).

**Fig 2:**
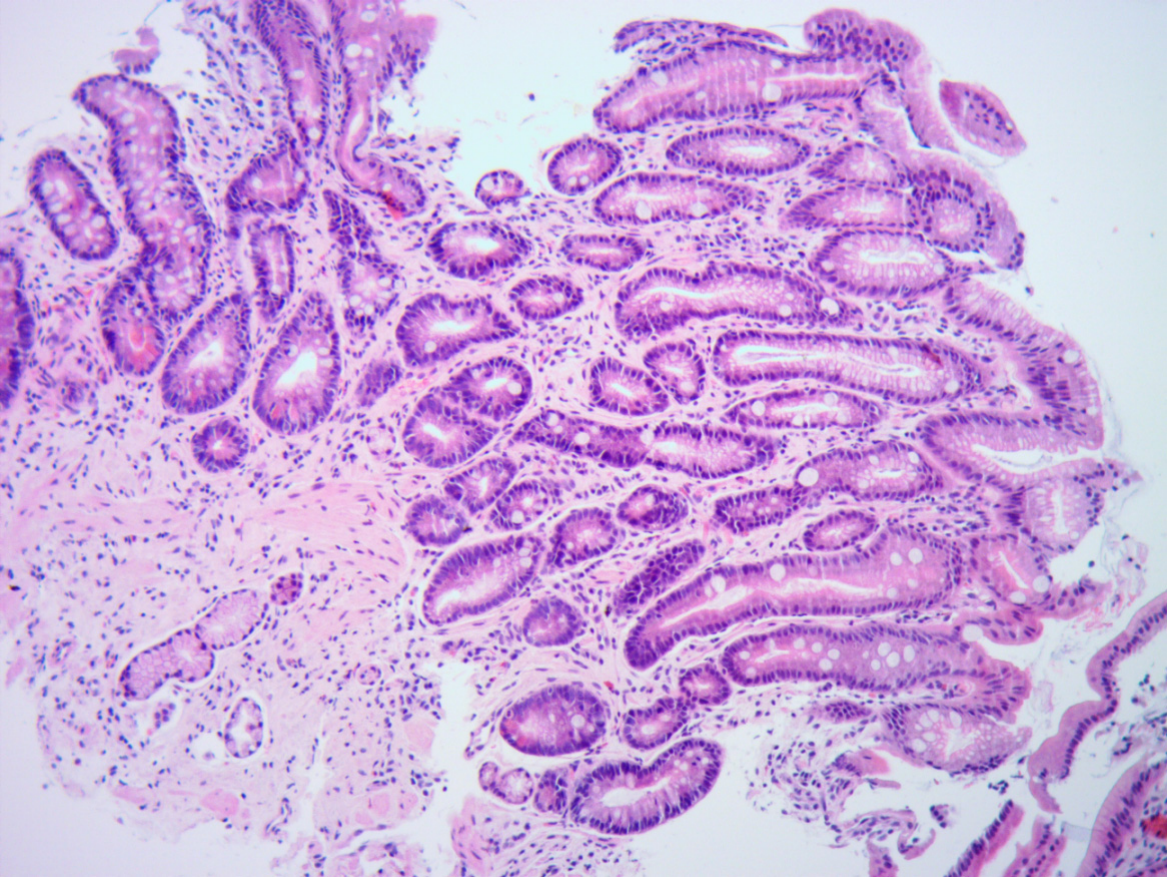
Multifocal atrophic chronic gastritis with intestinal metaplasia–MAG (H&E—original magnification 40x).

**Fig 3:**
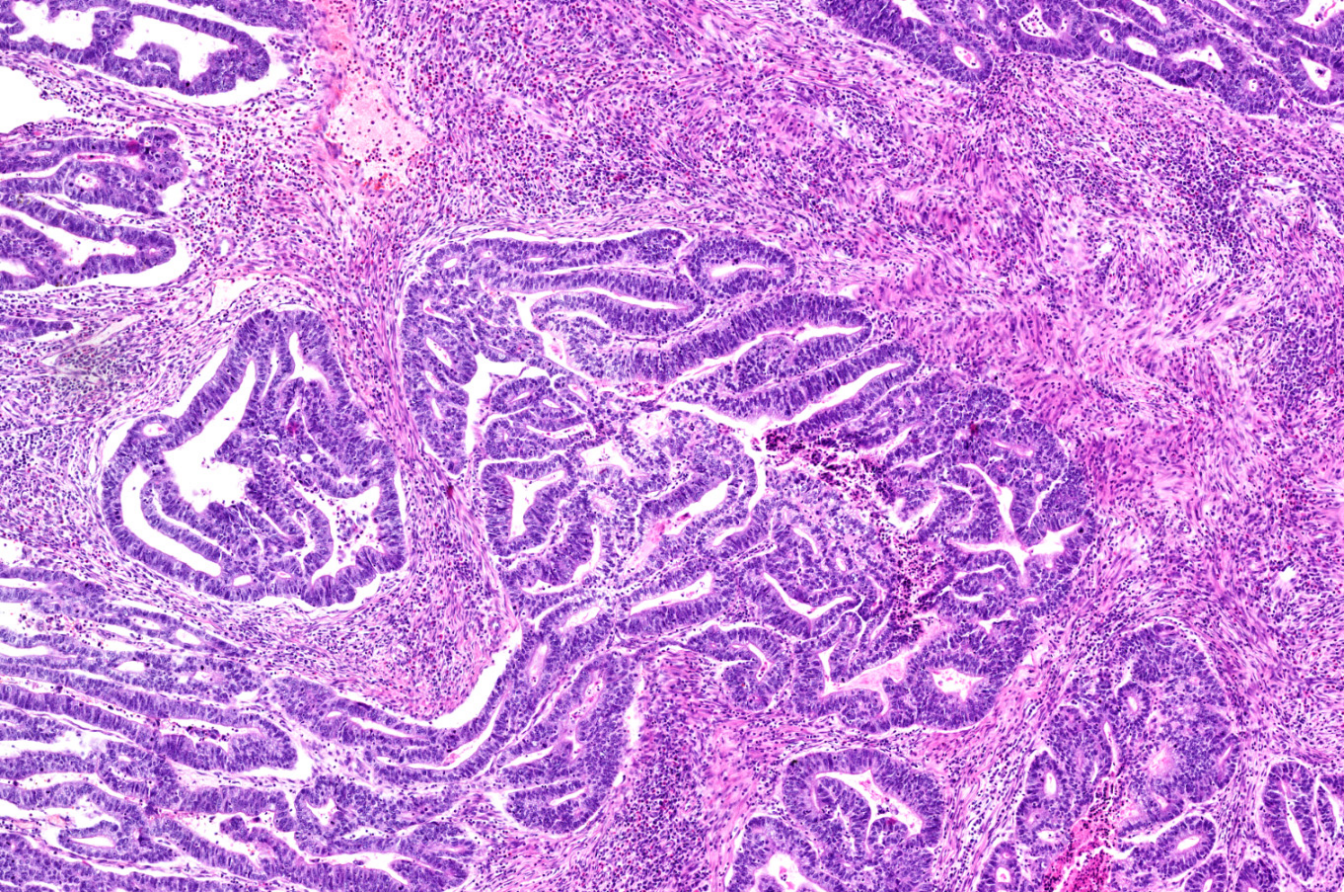
Gastric adenocarcinoma–GC (H&E—original magnification 40x).

Gastroscopic and histopathological evaluation allowed to distinguish 3 groups of patients. Group 1 included 18 patients (12 men and 6 women), 53 ± 8.1 years of age, carrying the diagnosis of non-atrophic gastritis (NAG), characterized by a mild or moderate intensity of chronic active inflammation but free of traits of atrophy or intestinal metaplasia (Fig 1). Group 2 included 9 patients (6 men and 3 women), 54 ± 7.6 years of age, in whom histopathological examination disclosed *H*. *pylori*-linked multifocal atrophic gastritis (MAG), characterized by moderate intensity of chronic active inflammation, with moderate multifocal atrophy and mild intestinal metaplasia (Fig 2). Group 3 included 15 patients (11 men and 4 women), 65 ± 4.8 years of age, with diagnosis of gastric adenocarcinoma (GC). In all the patients of group 3 an early stage of tumour development was diagnosed, T1 or T2 in TNM classification and low-grade (G1) (Fig 3).

Specimens of gastric mucosa were plated on Columbia agar (bioMerieux, Poland) with 7% sheep blood with antibiotic supplement (*Helicobacter pylori* Selective Supplement Dent SR, Oxoid). The incubation was conducted in microaerophilic conditions (Genbox microaer, bioMerieux) at 37°C for 4 to 7 days. The isolated *Helicobacter pylori* strains were identified on the basis of Gram staining as well as by production of urease, catalase, and oxidase. Moreover, the presence of *H*. *pylori* was identified in the tissue sections stained with Giemsa.

All the research protocols were reviewed and approved by the Ethics Committee by the University of Medical Sciences in Poznań, Poland.

### *H*. *pylori* DNA extraction and polymerase chain reactions

DNA was extracted from fresh cultures of *H*. *pylori* isolates using Swab kit (A&A Biotechnology, Poland). The isolation was conducted as recommended by the producer. The purified DNA was stored at the temperature of -20°C until further analyses were performed.

For detection of *cagA* gene, the diagnostic kit of PCR-*Helicobacter pylori* (DNA Gdańsk, Poland) was used. Amplification was conducted in line with instruction of the producer. The amplification was carried out in a Mastercycler pro S (Eppendorf, Germany). PCR product was subjected to electrophoresis in the 2% agar gel and the result was read after staining with ethidium bromide and visualised under UV light. Presence of PCR reaction in form of a product of 445 base pairs in size was accepted as the positive test result.

Detection of *vacA* gene was conducted using PCR method. The applied primers included vacaF: 5’-GCCGATATGCAAATGAGCCGC-3’ and vacaR: 5’-CAATCGTGTGGGTTCTGGAGC-3’) [19]. The reaction mixture contained 1 x PCR buffer, 0.2 mM dNTPs mix, 0.5 U Taq polymerase, 3 mM MgCl_2_, sterile distilled water (DNA Gdańsk, Poland), 25 pM of each primer (Sigma-Aldrich, Poland) and 1 μl of genomic DNA. The amplification was carried out in a Mastercycler pro S (Eppendorf, Germany) according to the following program: an initial denaturation step at 94°C for 10 min, followed by 35 cycles of denaturation at 94°C for 30 s, annealing at 66°C for 1 min, elongation at 72°C for 1 min and a final extension step at 72°C for 2 min. Amplified PCR products were resolved by electrophoresis using 2% agarose gels, stained with ethidium bromide and visualised under UV light. Presence of PCR reaction in form of a product of 678 base pairs in size was accepted as the positive test result.

### *H*. *pylori* RNA extraction and microarray assay

For RNA extraction *H*. *pylori* bacteria were used, originating from 72-h culture (conditions of the culture were given above) and adjusted to manifest density 0.5 in McFarland’s scale. Viability of all the grown up *H*. *pylori* strains amounted to ≥ 90%, which was evaluated under a fluorescence microscope (Nikon Eclipse E200) employing the Live⁄Dead Viability⁄Cytotoxicity Kit (Invitrogen, USA).

For every examined strain of *H*. *pylori* three independent experiments were conducted. Total RNA was isolated from *H*. *pylori* strains using Total RNA Mini Plus (A&A Biotechnology, Poland), following the manufacturer’s instruction. The quality of RNA was evaluated using a 2100 Bioanalyzer (Agilent Technologies, USA). RNA integrity number (RIN) was calculated for each sample using Agilent 2100 Expert software. Samples with RIN values acceptable for microarray study were stored at -80°C until needed.

In the studies *Helicobacter pylori SNT49* Gene Expression Microarrays 8×15K were used (Agilent Technologies, USA) [20]. The arrays were processed according to the Two-Colour Microarray Based Gene Expression Analysis protocol v. 6.7. RNA obtained from 10^6^ cells of every analysed *H*. *pylori* strain was amplified and labelled with fluorochromes using dye-swap approach. Labelling was performed with use of Low Input Quick Amp Kit (Two-Colour) (Agilent Technologies, USA) and purification of the labelled RNA was performed (Qiagen RNeasy Kit). Yield and specific activity were quantified using a NanoDrop ND1000 spectrophotometer (Thermo Scientific, USA). Labelled cRNA was fragmented and hybridized on microarrays in a balanced block design. Microarrays were incubated for 17 h at 65°C in an Agilent hybridization oven, washed in GE wash buffers and scanned using Agilent’s High Resolution C Microarray Scanner (Agilent Technologies, USA) at the settings recommended for the 8×15 K array format. The images obtained after scanning were extracted using Agilent Feature Extraction software v. 12 to obtain normalized data, depicting the intensity of fluorescence. The further bioinformatic analysis was performed using GeneSpring GX software v. 13 (Agilent Technologies, USA). The obtained data were deposited in ArrayExpress with the accession number E-MTAB-4229.

The obtained results represented an average value obtained in three-fold experiments per *H*. *pylori* strain and they were presented in relative fluorescence units (RFU). RFU are the arbitrary units in which fluorescence intensity is reported by the fluorescence imaging systems [21]. Fluorescence intensity of negative control in the studies amounted to 28 ± 9 RFU. Therefore, results above the cut-off value = 46 RFU (i.e. two standard deviations above mean value) were accepted as positive.

### Quantitative RT-PCR

Quantitative RT-PCR analysis was performed to verify results obtained in the microarray assays using a Eppendorf realplex4 thermal cycler. RT reactions were performed using Superscript III (Invitrogen, USA), according to the manufacturer’s instructions. The primer sequences for each gene are listed in Table 1 [22]. All sample and primer combinations were assessed in triplicates. We estimated relative gene expression normalized to the concentration of 16S rRNA. To amplify the cDNA, 2 μl of reverse-transcribed cDNA was subjected to PCR amplification in 20 μl containing 0.25 μM each primer, 10 μl of ready-to-use SensiFast SYBR No-ROX Kit (containing heat-activated DNA polymerase, dNTPs, MgCl_2_, SYBR^®^ Green I; Bioline, UK) and water. Each sample was run under the following PCR conditions for 40 cycles: an initial step at 95°C for 10 min, then 95°C for 20 s and 59°C for 1 min. PCR specificity and product detection was checked postamplification by examining the temperature-dependent melting curves of the PCR products.

**Table 1:** Primer sequences for real-time RT-PCR.

Gene	Forward primer sequence	Reverse primer sequence
16S rRNA	5’-GGAGGATGAAGGTTTTAGGATTG-3’	5’-TCGTTTAGGGCGTGGACT-3’
*virB11*	5’-CCTCTAAGGCATGCTACTGAAGAA-3’	5’-TCGCTAAATTGCTGCTCAAAA-3’
*vacA*	5’-TACAACAAACACACCGCAAAA-3’	5’-TGTAGCGATACCCCCAACCA-3’

### Statistical analysis

Data were analysed by using Statistica 10 (StatSoft, USA). The statistical analysis took advantage of Mann-Whitney test and Fisher’s exact test. Relationships with *P*-values higher than 0.05 were considered insignificant.

## Results

In group 1, among 18 obtained strains of *H*. *pylori*, in 12 (66.6%) of them, forming subgroup 1, *cagA* gene was identified (strains *cagA*^*+*^), while subgroup 2 was formed by 6 (33.3%) remaining isolates, which did not carry *cagA* gene (strains *cagA*^*-*^). In studies on strains isolated from patients with NAG no principal differences could be disclosed in gastric histopathology, which could be linked to manifestation of *cagA*^*+*^ and *cagA*^*-*^ strains. In all strains *vacA* gene was identified.

Among the subgroup 1, in all strains of *H*. *pylori* high expression of *cagA* gene and *virB/D* complex was demonstrated. The results are presented in Tables 2 and 3. However, the strains manifested moderate expression of *vacA* gene. In turn, in the subgroup 2 containing *cagA*^*-*^ strains expression of *cagA* and *virB/D* complex genes could not be detected in any of the isolates (the expression corresponded to values of the negative control); on the other hand a moderate expression of *vacA* was detected, the mean value of which is presented in Table 2.

**Table 2:** Expression in RFU (mean ± SD) of *cagA* and *vacA* genes in studied groups.

Gene	Group 1 (n = 18) Non-atrophic gastritis		Group 2 (n = 9) Atrophicgastritis	Group 3 (n = 15) Gastric adenocarcinoma
	Subgroup 1 (n = 12)	Subgroup 2 (n = 6)		
*cagA*	2943 ± 546	nd	3389 ± 699	9641 ± 1323 **
*vacA*	442 ± 72	496 ± 87	1476 ± 339 *	1569 ± 297 *

* significantly different than the result obtained in group 1 (p<0.05)

** significantly different that the results obtained in groups 1 and 2 (p<0.05)

nd—not detected

**Table 3:** Expression in RFU (mean ± SD) of *virB/D* complex genes in studied groups.

Gene	Group 1 (n = 18) Non-atrophic gastritis		Group 2 (n = 9) Atrophicgastritis	Group 3 (n = 15) Gastric adenocarcinoma
	Subgroup 1 (n = 12)	Subgroup 2 (n = 6)		
*virB4*	1206 ± 191	nd	1320 ± 157	1483 ± 315
*virB7*	1281 ± 206	nd	1213 ± 231	1360 ± 371
*virB8*	1241 ± 179	nd	1363 ± 158	1513 ± 327
*virB9*	1194 ± 226	nd	1155 ± 184	1288 ± 278
*virB10*	1257 ± 192	nd	1366 ± 172	1506 ± 355
*virB11*	1273 ± 160	nd	1195 ± 156	1433 ± 348
*virD4*	1063 ± 229	nd	1187 ± 247	1257 ± 320

nd—not detected

In group 2, *cagA* and *vacA* genes were identified in all the 9 strains. In parallel, a high expression of *cagA*, complex *virB/D* and *vacA* genes was demonstrated. The results are presented in Tables 2 and 3. Statistical analysis of the data showed that the mean value of *cagA* expression was around two-fold higher than mean expression of *vacA* (p<0.05).

In group 3, *cagA* and *vacA* genes were identified in all the 15 strains. In parallel, a superhigh expression was demonstrated of *cagA* gene and high expression of complex *virB/D* and *vacA* genes, with activity of *cagA* gene being around six-fold higher than that of *vacA* gene (p<0.05). The results are presented in Tables 2 and 3.

Statistical analysis of the data for studied groups demonstrated that *H*. *pylori* strains associated with GC (group 3) manifested the highest expression of *cagA* gene. In turn, *H*. *pylori* isolates originating from patients with MAG (group 2) did not differ from strains obtained from patients with NAG (forming subgroup 1) in expression of *cagA* gene (p>0.05). Also, no significant differences were detected in expression of *virB/D* complex genes between subgroup 1, group 2 and 3 (p>0.05). At the same time, mean results of *vacA* expression were the highest ones in strains obtained from patients with MAG and GC and they differed significantly from the results obtained on isolates in the group of patients with NAG (p<0.05).

## Discussion

In line with the paradigm of Correa [23], development of gastric cancer, already well documented in patients infected with *H*. *pylori*, involves a multistep process, determined by progressive injury to gastric mucosa [10, 24–26]. In the sequence of the *H*. *pylori*-induced process, the critical point involves transformation of the chronic active non-atrophic inflammation into atrophic gastritis with intestinal metaplasia. The metaplastic lesions may be followed by dysplasia, leading to gastric cancer. However, the multistage process is slow and linked to long-term presence of the pathogen. Therefore, the risk of gastric cancer among *H*. *pylori*-positive subjects is low and evaluated at 1–2% in Western countries [5]. In development of *H*. *pylori-*induced morbid lesions, principal significance is ascribed to its virulence factors, in particular to CagA and VacA [3, 27, 28]. Thus, it seems possible that activity of genes coding for the toxins determines a specific gastric pathology.

In this study we have obtained *H*. *pylori* isolates from patients with diagnoses of NAG, MAG, or GC, which permitted to perform for the first time comparative studies on expression of the virulence genes, including *cagA*, complex *virB/D* and *vacA*, in these three morbid units. In the studied isolates of *H*. *pylori* obtained from patients with NAG we have shown that the morbid process may be linked to both *cagA*^*+*^ strains (66.6% strains), and *cagA*^*-*^ strains (33.3% strains). Nevertheless, in the investigations we have not been able to note that those various strains were linked to specific differences in intensity or activity of chronic gastritis. The results contradict the earlier obtained data of other authors [29, 30], suggesting that *cagA*^*+*^ strains are associated with more severe gastritis. However, in the latter studies the isolates of *H*. *pylori* originated from patients with a variable spectrum of gastric pathology, and the *cagA* status of the pathogen was estimated on grounds of the produced CagA protein. In the other hand, in our studies the presented results have pertained only to the group of patients with diagnosis of non-atrophic gastritis, and the criterion of separating the isolates has involved presence of *cagA* gene. This may explain the divergent results.

All the *H*. *pylori cagA*^*+*^ strains from patients with NAG have been characterized by high expression of *cagA*^*+*^ and genes coding for secretory apparatus complex, *virB/D*. The analyzed isolates of *H*. *pylori cagA*^*+*^ and *cagA*^*-*^ from patients with NAG have manifested a moderate expression of *vacA* gene. The results are in line with earlier reports demonstrating that CagA protein plays only a minor role, if any, in induction of gastric inflammation [5, 31]. In parallel, it was pointed out that the process may be mediated by peptidoglycan molecules of bacterial cell wall, translocated directly into epithelial cells as a result intimate interaction with the T4SS structure [12, 32]. It was suggested also that structural components of the T4SS may additionally participate in induction of gastric inflammation [12]. On the other hand, the recently presented studies on an animal model documented a direct role of cytotoxin VacA in induction of gastric inflammation and damage [33]. Considering the above presented data, one may conclude that development of *H*. *pylori*-linked pathomechanism of NAG requires involvement of the pathogen’s secretory apparatus and/or its VacA toxin.

In turn, all the *H*. *pylori* strains originating from patients with MAG and GC carried *cagA* gene. However, they significantly differed in its expression: manifesting the high expression in isolates from patients with MAG and the almost three-fold higher hyperexpression in patients with GC (hypervirulent strains). In parallel, the strains have manifested high expression of genes coding for *virB/D* complex, which has been independent of *cagA* activity levels. The results may point to a lack of interaction between expression of *cagA* gene and that of genes coding for *virB/D* complex. Thus, the suggestion seems legitimate that translocation of CagA to host cells may be mediated not only by T4SS, but also with involvement of *cag*PAI-independent factors [12, 34]. Detection in this study of high expression of *cagA* in strains of *H*. *pylori* obtained from patients of subgroup 1 with NAG and in all patients with MAG, and also detection of the hypersecretion in isolates linked to GC, points to a significant role of CagA protein in infection with the pathogen. Nevertheless, expression of cagA in *H*. *pylori* has not differed between isolates originating from patients with NAG and those with MAG. The earlier studies documented that CagA, translocated to gastric epithelial cells, induced their morphological lesions, rearrangement of their actin cytoskeleton and variable phenotypes. Moreover, action of CagA is followed by activation of several signalling pathways, which may end up in neoplastic lesions [35–37]. Moreover, a direct effect of CagA protein on carcinogenetic process was demonstrated in *in vitro* experiments [38] and using animal models [39, 40]. In context of the data, the obtained by us results allow to conclude that CagA plays a significant role in establishment of chronic *H*. *pylori* infection, which may lead to development of pre-neoplastic lesions. However, progression of the lesions and development of GC may be determined by the very high expression of *H*. *pylori cagA* gene. The results are consistent with those obtained in *in vivo* studies conducted by Avilés-Jiménez et al. [22]. In parallel, our results remain in contrast to those *in vitro* results of the above quoted study. In contrast to the quoted study, however, we have used classical, optimal culture conditions for *H*. *pylori* [41], which may explain the differences obtained in the *in vitro* obtained results.

In analysis of *vacA* gene expression, we have demonstrated its moderate activity in H. pylori strains obtained from patients with NAG. At the same time, the vacA expression has been significantly higher in isolates originating from patients with MAG and GC. The results remain in perfect agreement with earlier publications, documenting significance of VacA cytotoxin in *H*. *pylori*-associated gastritis, ulceration, intestinal metaplasia and gastric cancer [33]. Moreover, they indicate that the elevated expression of vacA may be critical for transformation of non-atrophic gastritis into atrophic gastritis. In addition, our results indicate that development of *H*. *pylori*-induced carcinogenesis remains independent of a more pronounced increase in expression of *vacA*. The data allow to assume that VacA involves a long-lived protein, in contrast to the documented rapid degradation of CagA in gastric epithelial cells [42].

Thus, our results indicate that chronic active gastritis may be induced by both *cagA*^+^ *H*. *pylori* strains manifesting either high expression of genes coding for secretory apparatus complex, *virB/D*, and also by *cagA*^-^ strains with moderate expression of *vacA* gene. On the other hand, in progression of gastric pathology and in carcinogenesis linked to *H*. *pylori* a significant role is played by hypervirulent strains, characterized by very high expression of *cagA* and high activity of genes coding for the secretory apparatus complex, *vir B/D* and for *vacA*.

## References

[1] Cover TL, Blaser MJ. Purification and characterization of the vacuolating toxin from *Helicobacter pylori*. J Biol Chem. 1992; 267(15):10570–10575. 1587837

[2] Akopyants NS, Clifton SW, Kersulyte D, Crabtree JE, Youree BE, Reece CA, et al Analyses of the cag pathogenicity island of *Helicobacter pylori*. Mol Microbiol. 1998; 28(1):37–53. 9593295 10.1046/j.1365-2958.1998.00770.x

[3] Cover TL, Blanke SR. *Helicobacter pylori* VacA, a paradigm for toxin multifunctionality. Nat Rev Microbiol. 2005; 3(4):320–332. 15759043 10.1038/nrmicro1095

[4] Noto JM, Peek RM Jr. The *Helicobacter pylori* cag Pathogenicity Island. Methods Mol Biol. 2012; 921:41–50. 23015490 10.1007/978-1-62703-005-2_7 PMC3547679

[5] Kusters JG, van Vliet AH, Kuipers EJ. Pathogenesis of *Helicobacter pylori* infection. Clin Microbiol Rev. 2006; 19(3):449–490. 16847081 10.1128/CMR.00054-05 PMC1539101

[6] Warburton VJ, Everett S, Mapstone NP, Axon AT, Hawkey P, Dixon MF. Clinical and histological associations of cagA and vacA genotypes in *Helicobacter pylori* gastritis. J Clin Pathol. 1998; 51(1):55–61. 9577374 10.1136/jcp.51.1.55 PMC500433

[7] Höcker M, Hohenberger P. *Helicobacter pylori* virulence factors—one part of a big picture. Lancet. 2003; 362(9391):1231–1233. 14568748 10.1016/S0140-6736(03)14547-3

[8] Memon AA, Hussein NR, Miendje Deyi VY, Burette A, Atherton JC. Vacuolating cytotoxin genotypes are strong markers of gastric cancer and duodenal ulcer-associated *Helicobacter pylori* strains: a matched case-control study. J Clin Microbiol. 2014; 52(8):2984–2989. 10.1128/JCM.00551-14 24920772 PMC4136171

[9] Ki MR, Hwang M, Kim AY, Lee EM, Lee EJ, Lee MM, et al Role of vacuolating cytotoxin VacA and cytotoxin-associated antigen CagA of *Helicobacter pylori* in the progression of gastric cancer. Mol Cell Biochem. 2014; 396(1–2):23–32. 10.1007/s11010-014-2138-8 25038872

[10] Mégraud F, Bessède E, Varon C. *Helicobacter pylori* infection and gastric carcinoma. Clin Microbiol Infect. 2015; 21(11):984–990. 10.1016/j.cmi.2015.06.004 26086571

[11] Backert S, Selbach M. Role of type IV secretion in *Helicobacter pylori* pathogenesis. Cell Microbiol. 2008; 10(8):1573–1581. 10.1111/j.1462-5822.2008.01156.x 18410539

[12] Tegtmeyer N, Wessler S, Backert S. Role of the cag-pathogenicity island encoded type IV secretion system in *Helicobacter pylori* pathogenesis. FEBS J. 2011; 278(8):1190–1202. 10.1111/j.1742-4658.2011.08035.x 21352489 PMC3070773

[13] Selbach M, Moese S, Meyer TF, Backert S. Functional analysis of the *Helicobacter pylori* cag pathogenicity island reveals both VirD4-CagA-dependent and VirD4-CagA-independent mechanisms. Infect Immun. 2002; 70(2):665–671. 11796597 10.1128/iai.70.2.665-671.2002 PMC127714

[14] Christie PJ, Vogel JP. Bacterial type IV secretion: conjugation systems adapted to deliver effector molecules to host cells. Trends Microbiol. 2000; 8(8):354–360. 10920394 10.1016/s0966-842x(00)01792-3 PMC4847720

[15] Graham JE, Peek RM Jr, Krishna U, Cover TL. Global analysis of *Helicobacter pylori* gene expression in human gastric mucosa. Gastroenterology. 2002; 123(5):1637–1648. 12404238 10.1053/gast.2002.36589 PMC1361305

[16] Vannini A, Roncarati D, Spinsanti M, Scarlato V, Danielli A. In depth analysis of the *Helicobacter pylori* cag pathogenicity island transcriptional responses. PLOS ONE 2014; 9(6):e98416 10.1371/journal.pone.0098416 24892739 PMC4043881

[17] Dixon MF, Genta RM, Yardley JH, Correa P. Classification and grading of gastritis. The updated Sydney System. International Workshop on the Histopathology of Gastritis, Houston 1994. Am J Surg Pathol. 1996; 20(10):1161–1181. 8827022 10.1097/00000478-199610000-00001

[18] Bosman FT, Carneiro F, Hruban RH, Theise ND. WHO Classification of Tumours of the Digestive System. 4 th ed. IARC WHO, 2010.

[19] Tamer E, Wail H, Israr S, Sweidan W, Farraj MA. Determination of *Helicobacter pylori* virulence genes in gastric biopsies by PCR. ISRN Gastroenterology. 2013; (2013):ID 606258. 10.1155/2013/606258 PMC3649278 23691338

[20] Ali A, Naz A, Soares SC, Bakhtiar M, Tiwari S, Hassan SS, et al Pan-genome analysis of human gastric pathogen *H*. *pylori*: comparative genomics and pathogenomics approaches to identify regions associated with pathogenicity and prediction of potential core therapeutic targets. BioMed Res Int. 2015; ID 139580. 10.1155/2015/139580 PMC4325212 25705648

[21] Kane MD. Introduction to Gene Expression Profiling with DNA Microarray Technology In: Hardiman G. editor. Microarray Innovations: Technology and Experimentation. CRC Press, USA, 2009.

[22] Avilés-Jiménez F, Reyes-Leon A, Nieto-Patlán E, Hansen LM, Burgueño J, Ramos IP, et al In vivo expression of *Helicobacter pylori* virulence genes in patients with gastritis, ulcer, and gastric cancer. Infect Immun. 2012; 80(2):594–601. 10.1128/IAI.05845-11 22124657 PMC3264312

[23] Correa P. Human gastric carcinogenesis: a multistep and multifactorial process. First American Cancer Society Award Lecture on Cancer Epidemiology and Prevention. Cancer Res. 1992; 52(24):6735–6740. 1458460

[24] Konturek PC, Kania J, Konturek JW, Nikiforuk A, Konturek SJ, Hahn EG. *H*. *pylori* infection, atrophic gastritis, cytokines, gastrin, COX-2, PPAR gamma and impaired apoptosis in gastric carcinogenesis. Med Sci Monit. 2003; 9(7):SR53–66. 12883469

[25] Wróblewski LE, Peek RM Jr, Wilson KT. *Helicobacter pylori* and gastric cancer: factors that modulate disease risk. Clin Microbiol Rev. 2010; 23(4):713–739. 10.1128/CMR.00011-10 20930071 PMC2952980

[26] Jang BG, Kim WH. Molecular pathology of gastric carcinoma. Pathobiology. 2011; 78(6):302–310. 10.1159/000321703 22104201

[27] Argent RH, Thomas RJ, Letley DP, Rittig MG, Hardie KR, Atherton JC. Functional association between the *Helicobacter pylori* virulence factors VacA and CagA. J Med Microbiol. 2008; 57(Pt 2):145–150. 10.1099/jmm.0.47465-0 18201978

[28] de Bernard M, Josenhans C. Pathogenesis of *Helicobacter pylori* infection. Helicobacter. 2014; 19 Suppl 1:11–18. 10.1111/hel.12160 25167940

[29] Crabtree JE, Taylor JD, Wyatt JI, Heatley RV, Shallcross TM, Tompkins DS, Rathbone BJ. Mucosal IgA recognition of *Helicobacter pylori* 120 kDa protein, peptic ulceration, and gastric pathology. Lancet. 1991; 338(8763):332–335. 1677696 10.1016/0140-6736(91)90477-7

[30] Karita M, Blaser MJ. Acid-tolerance response in *Helicobacter pylori* and differences between cagA+ and cagA- strains. J Infect Dis. 1998; 178(1):213–219. 9652443 10.1086/515606

[31] Al-Ghoul L, Wessler S, Hundertmark T, Krüger S, Fischer W, Wunder C, et al Analysis of the type IV secretion system-dependent cell motility of *Helicobacter pylori*-infected epithelial cells. Biochem Biophys Res Commun. 2004; 322(3):860–866. 15336542 10.1016/j.bbrc.2004.07.199

[32] Viala J, Chaput C, Boneca IG, Cardona A, Girardin SE, Moran AP, et al Nod1 responds to peptidoglycan delivered by the *Helicobacter pylori* cag pathogenicity island. Nat Immunol. 2004; 5(11):1166–1174. 15489856 10.1038/ni1131

[33] Winter JA, Letley DP, Cook KW, Rhead JL, Zaitoun AA, Ingram RJ, et al A role for the vacuolating cytotoxin, VacA, in colonization and *Helicobacter pylori*-induced metaplasia in the stomach. J Infect Dis. 2014; 210(6):954–963. 10.1093/infdis/jiu154 24625807 PMC4136800

[34] Wessler S, Backert S. Molecular mechanisms of epithelial-barrier disruption by *Helicobacter pylori*. Trends Microbiol. 2008; 16(8):397–405. 10.1016/j.tim.2008.05.005 18619844

[35] Peek RM Jr, Blaser MJ. *Helicobacter pylori* and gastrointestinal tract adenocarcinomas. Nat Rev Cancer. 2002; 2(1):28–37. 11902583 10.1038/nrc703

[36] Amieva MR, Vogelmann R, Covacci A, Tompkins LS, Nelson WJ, Falkow S. Disruption of the epithelial apical-junctional complex by *Helicobacter pylori* CagA. Science. 2003; 300(5624):1430–1434. 12775840 10.1126/science.1081919 PMC3369828

[37] Hatakeyama M, Higashi H. *Helicobacter pylori* CagA: a new paradigm for bacterial carcinogenesis. Cancer Sci. 2005; 96(12):835–843. 16367902 10.1111/j.1349-7006.2005.00130.x PMC11159386

[38] Tsang YH, Lamb A, Romero-Gallo J, Huang B, Ito K, Peek RM Jr, et al *Helicobacter pylori* CagA targets gastric tumor suppressor RUNX3 for proteasome-mediated degradation. Oncogene. 2010; 29(41):5643–5650. 10.1038/onc.2010.304 20676134 PMC2980823

[39] Watanabe T, Tada M, Nagai H, Sasaki S, Nakao M. *Helicobacter pylori* infection induces gastric cancer in mongolian gerbils. Gastroenterology. 1998; 115(3):642–648. 9721161 10.1016/s0016-5085(98)70143-x

[40] Kodama M, Murakami K, Nishizono A, Fujioka T. Animal models for the study of *Helicobacter*-induced gastric carcinoma. J Infect Chemother. 2004; 10(6):316–325. 15614454 10.1007/s10156-004-0353-z

[41] Mégraud F, Lehours P. *Helicobacter pylori* detection and antimicrobial susceptibility testing. Clin Microbiol Rev. 2007; 20(2):280–322. 17428887 10.1128/CMR.00033-06 PMC1865594

[42] Ishikawa S, Ohta T, Hatakeyama M. Stability of *Helicobacter pylori* CagA oncoprotein in human gastric epithelial cells. FEBS Lett. 2009; 583(14):2414–2418. 10.1016/j.febslet.2009.06.043 19560464

